# Fungal endophytes of Brassicaceae: Molecular interactions and crop benefits

**DOI:** 10.3389/fpls.2022.932288

**Published:** 2022-08-05

**Authors:** Jorge Poveda, Sandra Díaz-González, María Díaz-Urbano, Pablo Velasco, Soledad Sacristán

**Affiliations:** ^1^Institute for Multidisciplinary Research in Applied Biology (IMAB), Universidad Pública de Navarra (UPNA), Pamplona, Spain; ^2^Centro de Biotecnología y Genómica de Plantas (CBGP, UPM-INIA/CSIC), Universidad Politécnica de Madrid (UPM), Instituto Nacional de Investigación y Tecnología Agraria y Alimentaria (INIA/CSIC), Madrid, Spain; ^3^Group of Genetics, Breeding and Biochemistry of Brassicas, Misión Biológica de Galicia (MBG), Spanish National Research Council (CSIC), Pontevedra, Spain; ^4^Departamento de Biotecnología-Biología Vegetal, Escuela Técnica Superior de Ingeniería Agronómica, Alimentaria y de Biosistemas, Universidad Politécnica de Madrid (UPM), Madrid, Spain

**Keywords:** glucosinolates, molecular dialog, *Arabidopsis*, *Brassica*, plant growth promotion, abiotic stress tolerance, biological control agent, mycorrhiza

## Abstract

Brassicaceae family includes an important group of plants of great scientific interest, e.g., the model plant *Arabidopsis thaliana*, and of economic interest, such as crops of the genus *Brassica* (*Brassica oleracea, Brassica napus*, *Brassica rapa*, etc.). This group of plants is characterized by the synthesis and accumulation in their tissues of secondary metabolites called glucosinolates (GSLs), sulfur-containing compounds mainly involved in plant defense against pathogens and pests. Brassicaceae plants are among the 30% of plant species that cannot establish optimal associations with mycorrhizal hosts (together with other plant families such as Proteaceae, Chenopodiaceae, and Caryophyllaceae), and GSLs could be involved in this evolutionary process of non-interaction. However, this group of plants can establish beneficial interactions with endophytic fungi, which requires a reduction of defensive responses by the host plant and/or an evasion, tolerance, or suppression of plant defenses by the fungus. Although much remains to be known about the mechanisms involved in the Brassicaceae-endophyte fungal interaction, several cases have been described, in which the fungi need to interfere with the GSL synthesis and hydrolysis in the host plant, or even directly degrade GSLs before they are hydrolyzed to antifungal isothiocyanates. Once the Brassicaceae-endophyte fungus symbiosis is formed, the host plant can obtain important benefits from an agricultural point of view, such as plant growth promotion and increase in yield and quality, increased tolerance to abiotic stresses, and direct and indirect control of plant pests and diseases. This review compiles the studies on the interaction between endophytic fungi and Brassicaceae plants, discussing the mechanisms involved in the success of the symbiosis, together with the benefits obtained by these plants. Due to their unique characteristics, the family Brassicaceae can be seen as a fruitful source of novel beneficial endophytes with applications to crops, as well as to generate new models of study that allow us to better understand the interactions of these amazing fungi with plants.

The beneficial symbiotic relationships between fungi and plants are firstly based on the fungal ability to evade host plant defenses and to engage in a mutualistic interaction with the plant. In this context, the beneficial interaction between plants of the Brassicaceae family and endophytic fungi represents a remarkable object of study with specific characteristics. In this review, we first describe the Brassicaceae family and its distinguishing features, the mechanisms that fungal endophytes may use to overcome plant defenses and colonize the tissues of Brassicaceae hosts, and, finally, the benefits that Brassicaceae plants may obtain from fungal endophytes and the known mechanisms involved.

## Brassicaceae family: Important crops and model plants

**FIGURE 1:**
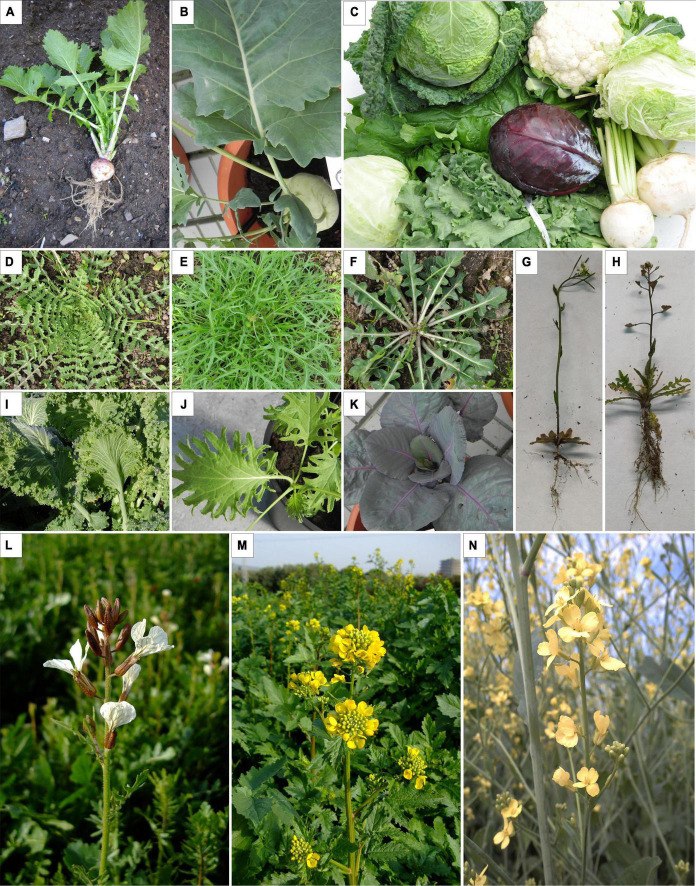
Composition of images of species belonging to the Brassicaceae family. **(A)** *Brassica rapa* (turnip), **(B)** *Brassica oleracea* var. gongylodes (Kohlrabi). **(C)** Different vegetable products belonging to the *Brassica* genus (cauliflower, cabbage, turnip…), **(D)** *Brassica barrelieri*, **(E)** *B. rapa* subsp. *Nippossinica*, **(F)** *Brassica gravinae*, **(G)** *Arabidopsis thaliana*, **(H)** *Capsella* sp., **(I)** *B. oleracea* (cabbage), **(J)** *Brassica juncea*, **(K)** *B. oleracea* subsp. *capitata* (red cabbage), **(L)** *Eruca vesicaria*, **(M)** *Sinapis alba*, **(N)** *Brassica carinata*.

The Brassicaceae (Cruciferae) family is one of the most economically and scientifically relevant families of dicotyledons. Generally, plants in the Brassicaceae family are herbs, herbaceous woody climbers, shrubs, or small trees. The roots can be conical, fusiform, or napiform and usually store food. The floral structure is maintained throughout the family, with four petals oriented in the shape of a cross, giving the name “Cruciferae” to the family. The fruits are usually siliques or siliqua types (Jabeen, 2020). Brassicaceae crops were probably domesticated in the Neolithic period in various regions of Eurasia and North Africa. Nowadays, these crops are consumed as vegetables, condiments, or oils, and, in addition to nourishing animals and humans, Brassicaceae plants have also been used for medical purposes, research, ornamentation, and bio-fumigation. The enormous diversity in its uses may be because this family has more than 321 genera and 3,660 species (Raza et al., 2020). Some of the genera that stand out in this family are *Arabidopsis, Brassica, Capsella, Crambe, Eruca, Diplotaxis, Moricandia, Orychophragmus*, or *Pachycladon* (Figure 1). However, the most important genus from an economic perspective due to its role in agriculture is Brassica. As of today, the genome sequences of more than 20 Brassicaceae species are available (Chen et al., 2022), including those of several *Brassica* crops (He et al., 2021).

The *Brassica* genus contains 37 species, which include the most relevant crops of the Brassicaceae family. The three most renowned species are *Brassica oleracea*, *Brassica napus*, and *Brassica rapa*. These species are strongly related to each other, and their phylogenetic relationships are collected in the Triangle of U theory U (1935), based on genomic and cytological studies (Allender and King, 2010). This theory states that the three diploid species, *Brassica nigra* (2*n* = 16, genome BB), *B. oleracea* (2*n* = 18, genome CC), and *B. rapa* (2*n* = 20, genome AA), underwent a two-by-two interspecific hybridization and generated the amphidiploids *B. napus* (AACC, 2*n* = 38), *Brassica juncea* (AABB, 2*n* = 36), and *Brassica carinata* (BBCC, 2*n* = 34).

*B. oleracea, B. napus*, and *B. rapa* are cultivated practically throughout the whole world (Jabeen, 2020; Raza et al., 2020). *B. oleracea* is used as a vegetable (such as cabbage, kale, brussels sprout, broccoli, or cauliflower), oil, and fodder crop. In 2020, the production of cabbage and other *B. oleracea* vegetables was approximately 70 M tons, with China and India being the main producers. It can be highlighted that only the production of broccoli and cauliflower reached the 25 M tons, with a gross production of 15 M US$ (FAOSTAT, 2022). The most important crop of *B. napus* is canola, rapeseed or colza, mostly used for oil production. Indeed, *B. napus* is the third source of vegetable oil worldwide, after soybeans and palm (Friedt et al., 2018), entailing a gross production of 36M US$ in 2018 (FAOSTAT, 2022). *B. rapa* can also be cultivated for its oil, although in some regions it is more popular for the consumption of its foliar parts or its roots, such as turnip, Asian greens, or pak choy (Raza et al., 2020). *B. nigra*, *B. juncea*, and *B. carinata* are used primarily as condiments, although they may be used for their oil and as leafy vegetables in certain regions (Rakow, 2004). In addition to the *Brassica* genus, species within *Sinapis, Camelina, Crambe, Eruca*, and *Raphanus* genera are also relevant in the oil industry due to their use as biofuel, lubricant, or for human and animal consumption.

Besides its importance as crops, the Brassicaceae family has a remarkable relevance in research, and this is mainly due to the species *Arabidopsis thaliana*, one of the best-studied model organisms. *A*. *thaliana* was the first plant species to have its genome sequenced and is essential in the study of plant genetics and physiology (Koornneef and Meinke, 2010). Native to Europe, Asia, and some regions of Africa, it is currently present throughout the world (Jabeen, 2020). Besides *A. thaliana*, other Brassicaceae plants used in research are, for example, *A. helleri*, which is useful for studying heavy metal contamination (Jabeen, 2020) or *Capsella* spp., which stand out for their wide distribution and high population densities (Mitrović et al., 2020).

## Glucosinolates: Relevant defense and nutraceutical compounds in the Brassicaceae family

Plants in the Brassicaceae family contain several bioactive chemicals that can act in beneficial or harmful ways, including glucosinolates, other sulfur-containing compounds, and erucic acid. Glucosinolates (GSLs) are amino acid-derived natural plant products found exclusively throughout the Capparales order, as well as the major class of secondary metabolites found in the family Brassicaceae. Approximately 150 GSLs have been identified (Halkier and Gershenzon, 2006; Blažević et al., 2020). Species in the Brassicaceae family typically produce between 30 and 40 different GSLs. GSL types and concentrations within the plants depend on the species and variety, and are influenced by the environmental conditions under which the plant is growing (Czerniawski et al., 2021). The hydrolytic breakdown products of GSLs, especially isothiocyanates (ITCs), have different biological effects. The most recognized and studied during the last decade is the biocidal effect, including antifungal, antimicrobial, herbicidal, and even insecticidal and nematicidal effects (Prieto et al., 2019). There is evidence that the distribution of GSLs throughout the plant tissues may be linked to the probability of a plant organ being attacked by pathogens or pests (Poveda et al., 2020b). However, it should be mentioned that family-adapted herbivores may not be negatively affected by these compounds. *Delia radicum*, *Pieris rapae*, or *Plutella xylostella* recognize certain GSLs that act as signals to stimulate oviposition (Städler and Reifenrath, 2009). GSL degradation products are also highly recognized for their chemopreventive, anti-inflammatory and antimutagenic effects, which have a protective function against diseases like cancer, atherosclerosis, degenerative heart diseases, glaucoma, etc. (Prieto et al., 2019). In addition, GSL degradation products provide organoleptic characteristics, associated with the pungent and bitter taste and the sulfurous aroma of the Brassicaceae crops.

**FIGURE 2:**
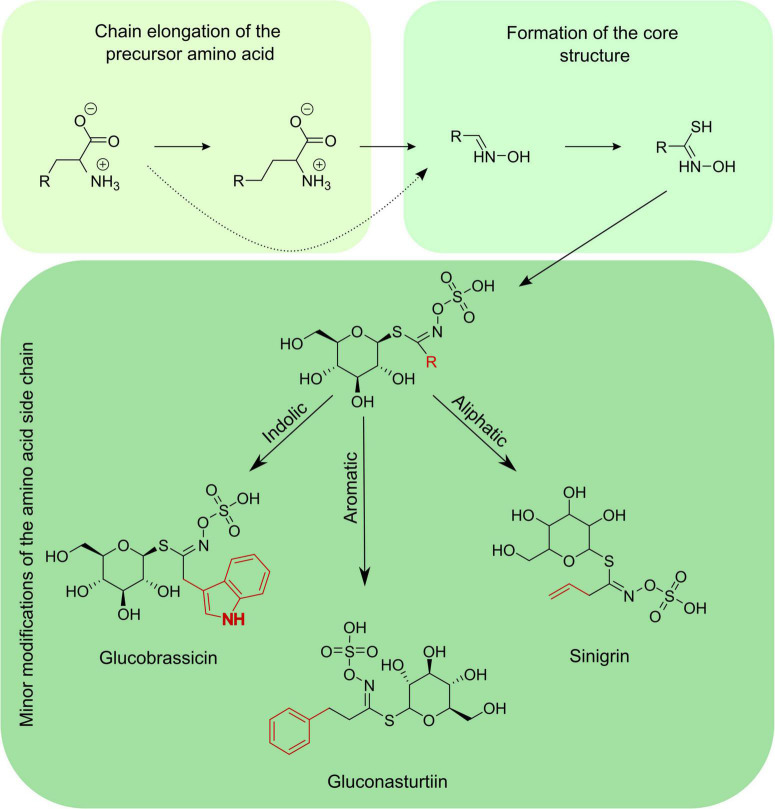
Diagram showing the three stages of the GSL biosynthetic pathway: chain elongation of the precursor amino acid, formation of the GSL core structure, and minor modifications of the amino acid side chain process.

The GSLs consist of a β-D-glucopyranose residue linked to a hydroximinosulfate ester by a sulfur bridge, plus an R-group. The R-group is derived from one of the eight amino acids and can be aliphatic (alanine, leucine, isoleucine, methionine, or valine), aromatic (phenylalanine or tyrosine), or indolic (tryptophan) (Halkier and Gershenzon, 2006). The GSLs biosynthesis occurs through three independent stages: first, there is the chain elongation of the precursor amino acid; the second stage is the formation of the core structure; and the third one is the secondary modifications of the amino acid side chain process (Figure 2). These changes in structure due to secondary modifications, side-chain elongations, substitutions to the side chain, or esterifications of the thioglucose moiety are responsible for all GSL structures known to date (Sønderby et al., 2010). GSLs are constitutively produced, but differences in quantity and type of GSLs occur between different parts and organs of the plants (Poveda et al., 2020b; Czerniawski et al., 2021). Also, GSL biosynthesis and hydrolysis can be modulated by biotic and abiotic stimuli (Bones and Rossiter, 2006; Bednarek et al., 2011). Despite the high amount of knowledge accumulated during the last decades on GSL, aspects of the temporal and spatial regulation of GSL biosynthesis, accumulation, and hydrolysis still need much investigation (Czerniawski et al., 2021).

The enzymes that catalyze the hydrolysis of GSLs to ITCs and other compounds are the myrosinases. Specifically, they catalyze the first step of bioactivation of GSLs, the hydrolysis of their thioglycosidic bond. Myrosinases are thioglucosidases (thioglucoside glucohydrolases, EC 3.2.1.147) of glycoside hydrolase family I, along with other b-glycosidases. Usually, they are composed of two identical polypeptides, which are glycosylated, resulting in a native molecular weight of 120–150 kDa of the dimeric proteins. Myrosinases accept different types of GSLs as substrates, but they differ in their affinity and conversion efficiency. GSL-containing species often possess several myrosinase isoforms with preferences for different substrates or specific to organs, tissues, or developmental stages (Wittstock et al., 2016). Depending on the parent GLS, the hydrolysis conditions (pH, temperature), presence of Fe^2+^ ions, and additional protein factors, the aglucon is converted into different classes of degradation products, thus, embracing any of the following: ITCs, thiocyanates, nitriles, epithionitriles (EPTs), hydroxynitriles, oxazolidine-2-thiones, or indoles. Myrosinases are often physically separated from their substrates at a cellular or subcellular level (Nakano et al., 2017; Parchem et al., 2020). Depending on the type of myrosinases, these can be present in myrosin cells, whose distribution differs in individual plant parts and also depends on the stage of development (Andréasson et al., 2001) or in endoplasmic reticulum (ER) bodies (Nakano et al., 2014). ER bodies are distributed in the root, hypocotyl, and cotyledons, but absent in leaves, and contain PYK10-like myrosinases (Yamada et al., 2020). It has been shown that ER body’s number or shape are altered in the presence of the defense-related phytohormone methyl jasmonate or wounds (Nakano et al., 2014; Gotté et al., 2015). Tissue damage caused by phytophagous insects initiates myrosinase-catalyzed glucosinolate hydrolysis and generation of ITCs and other toxic compounds that defend the plant against attack (Barth and Jander, 2006), and similar mechanisms may also be involved in defense against pathogens (Tierens et al., 2001). However, other GSL hydrolysis mechanisms are independent of cellular destruction, such as the indole glucosinolates (IGs) breakdown that takes place in living plant cells and is initiated by the atypical peroxisome associated myrosinase PEN2, which accumulates at pathogen contact sites during pre-invasive defense responses (Bednarek et al., 2009). The metabolic pathway triggered by PEN2 is needed for broad-spectrum defense against invasion and growth of a diverse range of pathogenic fungi (Hiruma, 2019). Also, besides the supposed direct toxic effect on pests and pathogens, the metabolic pathway of IGs is related to signaling and activation of evolutionarily conserved immune responses in the plant (Pastorczyk and Bednarek, 2016).

## Plant–fungus interactions in Brassicaceae: From pathogens to endophytes

Brassicaceae plants, like all other plant species, can establish a wide variety of relationships with fungi, ranging from negative interactions with pathogens to positive associations with beneficial fungi (Table 1). Besides, interactions can transit among neutral (commensals), negative (parasitic), or positive (mutualistic) depending on the environmental conditions or the genetic variation in both fungus and plant host (Rai and Agarkar, 2016; Zeilinger et al., 2016). The interactions with pathogens have been the most studied and characterized ones for many years, due to the economic impact of fungal diseases on crops (Poveda et al., 2020a; Thoms et al., 2021). However, today it is known that a large fraction of the fungal interactions with plants is mutualistic and results in beneficial outcomes for both partners (Kivlin et al., 2013; Martin et al., 2017; Strobel, 2018). Although fungal pathogens are still a major threat to crops worldwide, many fungal species might be now regarded as allies, rather than enemies, to improve the resilience of the plant to the changing environment and sustainably increase crop productivity (Lugtenberg et al., 2016; Poveda et al., 2021; Pozo et al., 2021).

**TABLE 1:** Definitions box.

Concept	Definition	References
Symbiosis	The living together of two different kinds of organisms that may, but not necessarily, benefit each organism.	D’Arcy et al., 2001
Mutualism	A relationship between organisms in which both benefit.	D’Arcy et al., 2001
Parasite	An organism that lives in intimate association with another organism on which it depends for its nutrition; not necessarily a pathogen (contrasts with saprophyte)	D’Arcy et al., 2001
Pathogen	A disease-producing organism or biotic agent.	D’Arcy et al., 2001
Endophyte	Microorganism that naturally colonizes internal plant tissues without causing disease symptoms for at least part of its life cycle.	Hardoim et al., 2015

The rich variety of mutualistic plant-fungal interactions includes both mycorrhizal and endophytic fungi (Rodriguez et al., 2009; Hardoim et al., 2015; Brundrett and Tedersoo, 2018; Genre et al., 2020). Mycorrhizae are the best-known plant–fungus mutualisms, especially those classified as arbuscular mycorrhiza (AMF) (Parniske, 2008; Miyauchi et al., 2020). Mycorrhizal fungi are characterized by the formation of specific structures in the host plant (i.e., vesicles or arbuscules), establishing fine-tuned relationships, in which the plant receives phosphorus and other mineral nutrients in return for carbohydrates and lipids (Gutjahr and Parniske, 2013; Cosme et al., 2018; Genre et al., 2020). Besides, mycorrhiza can also confer plant resistance to abiotic and biotic stresses (Rivero et al., 2018; Sanmartín et al., 2020). However, about 30% of vascular plant species cannot establish optimal mycorrhizal associations. Members of the Brassicaceae family are found within this group of non-mycorrhizal plants, including the model plant *A. thaliana* and relevant crops, such as oil-seed rape (*B. napus*), cabbage (*B. oleracea*), or turnip (*B. rapa*) (Cosme et al., 2018). Phylogenetic analyses within the Brassicaceae family point to the loss of essential genes for mycorrhizal symbiosis that occurred independently during non-mycorrhizal lineages’ evolution (Delaux et al., 2014; Bravo et al., 2016). Indeed, several Brassicaceae species form optimal mycorrhizae (Orłowska et al., 2002; Vogel-Mikuš et al., 2006), and some others considered non-mycorrhizal, such as Arabidopsis or *B. napus*, are colonized by mycorrhizal fungi to some extent, forming rudimentary structures, which, however, are not fully functional or even detrimental for the host plant (Tommerup, 1984; Glenn et al., 1985; Demars and Boerner, 1996; Veiga et al., 2013). The amount and diversity of GSL may be an important factor in preventing mycorrhization (Vierheilig et al., 2000; Anthony et al., 2020). These authors showed that Brassicaceae crops lacking myrosinase enzymes were root-colonized by mycorrhizal fungi, pointing out the key role of these enzymes and their hydrolysis products in the evolutionary loss of mycorrhization by Brassicaceae plants (Vierheilig et al., 2000). It has also been shown that GSLs protect Arabidopsis from the detrimental colonization by mycorrhiza forming fungi (Anthony et al., 2020). Since both, the loss of symbiotic genes and the presence of particular GSLs pathways, correlate with the lack of mycorrhization ability, a relationship between the three features in Brassicaceae can be hypothesized, although this possible link has not been proven yet (Hiruma et al., 2018).

Besides mycorrhiza, fungal endophytes have emerged as an alternative group of fungi conferring multiple benefits to plant hosts (Yan et al., 2019; Sarkar et al., 2021). Fungal endophytes are a highly diverse group of fungi that naturally colonize internal plant tissues without causing disease symptoms for at least part of their life cycle (Rodriguez et al., 2009; Hardoim et al., 2015) (Table 1). Many of the plant-endophyte interactions are mutualistic, with the exchange of benefits between the host and the endophyte. Thus, the endophyte can promote plant growth, facilitate plant adaptation and increase tolerance to biotic and abiotic stresses, while the host plant provides shelter and nutrients to the fungus (Poveda et al., 2021; Sarkar et al., 2021). Endophytic fungi do not form the morphologically complex structures within host plant cells typical of mycorrhiza (Peterson et al., 2008) and, also in contrast with these obligate symbionts, many of the fungal endophytes are axenically cultivable (Mesny et al., 2021). However, fungal endophytes have been much less studied than mycorrhiza so, in most the cases, the mechanisms behind their interactions with the plant are not well-understood (Card et al., 2015; Chitnis et al., 2020; Orozco-Mosqueda and Santoyo, 2021). The association with fungal endophytes seems to be universal across the plant kingdom, being found in all surveyed plants and environments (Rodriguez et al., 2009; Aamir et al., 2020). Thus, fungal endophytes acquire special relevance for non-mycorrhizal plants (i.e., many of the Brassicaceae species) as they can compensate for the lack of ability for mycorrhization by providing counterpart benefits (Hiruma et al., 2018).

During the last decade, several cultivation-dependent and independent surveys have described the composition and diversity of fungal endophytes in wild populations of Arabidopsis and other wild Brassicaceae species across different environments (Junker et al., 2012; García et al., 2013; Keim et al., 2014; Glynou et al., 2016; Almario et al., 2017; Thiergart et al., 2020). Regarding Brassicaceae crops, fungal endophytes have been surveyed and isolated from turnip (Liang et al., 2017), rapeseed (Glynou et al., 2018), kale (Poveda et al., 2020c), Chinese cabbage, radish, or white cabbage (Chen et al., 2020), as some examples. These works highlight the high diversity of fungal endophytes hosted by them, comparable to other hosts. Besides, the composition of the endophytic fungal assemblages of these hosts, in general, is not very different from other non-Brassicaceae hosts (Hiruma et al., 2018; Mesny et al., 2021). Indeed, climate or seasonal and soil factors seem to be stronger drivers of mycobiota composition than host genotype (García et al., 2013; Glynou et al., 2016; Urbina et al., 2018; Thiergart et al., 2020), even when comparing among different Brassicaceae (Glynou et al., 2018; Chen et al., 2020; Maciá-Vicente et al., 2020) or between Brassicaceae and non-Brassicaceae species, as in the canola (*B. napus*) versus wheat comparison (Schlatter et al., 2019). However, other works suggest a reduced diversity of soil communities exposed to canola-derived GLSs (Lay et al., 2018; Siebers et al., 2018). Even some works point to a higher frequency of particular taxa, such as species in the order Helotiales, associated with Brassicaceae hosts (Almario et al., 2017; Chen et al., 2020; Maciá-Vicente et al., 2020). Therefore, more studies comparing Brassicaceae and non-Brassicaceae hosts are needed to fully ascertain if the features of Brassicaceae metabolites condition microbiota recruitment, and the extent of this effect on the composition and performance of the endophytic mycobiota.

## How do fungal endophytes interact with Brassicaceae?

Like any other microorganism intending to enter the plant, fungal endophytes need to evade the plant detection and immunity system (Teixeira et al., 2019). The first layer of the plant detection system is the cell-surface pattern recognition receptors (PRRs) that can recognize microbe molecular patterns conserved among practically all microorganisms that reach the plant (called MAMPS). PRRs transduce signals that activate defense reactions encompassed within the so-called pattern-triggered immunity (PTI) (Zipfel and Oldroyd, 2017; Albert et al., 2020). To evade these plant defense reactions and enter into the plant, microorganisms may use effectors that, in turn, can be detected by the second layer of plant receptors (mainly, but not only, nucleotide-binding leucine-rich repeat – NRL- receptors), touching off the second layer of defense reactions, the effector-triggered immunity (ETI, Jones and Dangl, 2006). The number and structural features of PRRs and NLR receptors in Brassicaceae varies, as it is in other plant families (Dufayard et al., 2017; Gao et al., 2018). Brassicaceae species are not among those with higher numbers of these receptors, but many of them, mostly belonging to *A. thaliana*, are among the best known and characterized (Ngou et al., 2022). Indeed, in the study by Ngou et al. (2022), 28 of the 59 PRRs with known ligands have been characterized in *A. thaliana*, and one in *B. napus*. Twelve of these receptors, including the one in *B. napus*, specifically perceive fungal cell wall-derived MAMP ligands, such as chitin and oligogalacturonides (OGs) (Brutus et al., 2010; Gong et al., 2020), and apoplastic effectors or other proteinaceous elicitors, including phytocytokine-like peptides (Long et al., 2011; Zhang L. et al., 2014; Rhodes et al., 2021). Regarding NLR, Brassicaceae species tend to express less of these receptors in roots, in contrast with most other plant species (Munch et al., 2018). This particular feature suggests that Brassicaceae roots do not depend greatly on NLRs to defend against microbial attempts to enter (Hiruma et al., 2018). Instead, constitutive levels and profiles of GLS and other secondary components in Brassicaceae roots may be important defense components and might play a relevant role in the recruitment of root microbiota (Szűcs et al., 2018; Plaszkó et al., 2021).

The two-tiered immune detection and response model system is mostly based on knowledge on the interactions of plants with pathogens (Jones and Dangl, 2006; Ngou et al., 2022). More recently, it became clear that the recognition mechanisms also hold for beneficial microorganisms, such as mycorrhizae, although with some differences (Zipfel and Oldroyd, 2017; Thoms et al., 2021). As it happens with pathogenic and mycorrhizal fungi, plant receptors should recognize molecular patterns from fungal endophytes, eliciting immune responses. Indeed, different works show that gene expression changes in Arabidopsis at the early stages of endophytic fungal infection are comparable to those induced by pathogens (Fesel and Zuccaro, 2016; Guo et al., 2021). Plant recognition of pathogenic or mycorrhizal fungi triggers local and systemic defense responses, in which SA- and JA/ET phytohormones-dependent pathways are mainly involved (Pieterse et al., 2009, 2014). In the same way, fungal endophytes also elicit changes in these phytohormones and have mechanisms to manipulate this early plant defensive response. Arabidopsis colonization by *Serendipita* spp. endophytes is followed by an increase in JA and a reduction in SA levels (Jacobs et al., 2011; Lahrmann et al., 2015). SA- and JA- related genes are also downregulated in *A. thaliana* after 24 h of incubation in the presence of *Trichoderma harzianum* (Morán-Diez et al., 2012). Indeed, *T. harzianum* requires local suppression of SA-related local defenses for root penetration and colonization (Poveda et al., 2019b), in analogy with the defense suppression necessary for mycorrhiza formation in host plants (Pozo and Azcón-Aguilar, 2007). Thus, it has been documented that *T. harzianum* inoculation allowed root colonization of co-inoculated arbuscular mycorrhizal fungi (AMF) in Arabidopsis and rapeseed (Poveda et al., 2019b), indicating that modifications in defense responses by *T. harzianum* in these non-mycorrhizal hosts “opened the door” to AMF, exerting a synergistic effect between both fungal symbionts and the plant. Remarkably, an intact innate immune system is necessary for the beneficial effect of endophytic fungi, preventing its excessive growth and pathogenic behavior. Arabidopsis SA accumulation-deficient mutants are unable to prevent entering the root vascular system and pathogenic outcome in the interaction with *T. harzianum* (Alonso-Ramírez et al., 2014), and plant growth is not promoted or even inhibited by the fungus in ethylene response mutants (Camehl et al., 2010). In this sense, Brassicaceae-specific defense-related GSLs are very important components in the interaction with endophytes, as shown with *Colletotrichum tofieldiae* and *Serendipita* spp. endophytes, which fail to induce beneficial effects in Arabidopsis mutants in IGs synthesis pathways (Lahrmann et al., 2015; Hiruma et al., 2016). However, the role of these compounds is ambiguous and may change depending on the endophyte. Arabidopsis mutants in IG synthesis were better colonized by *Trichoderma* spp. root endophytes, increasing silique production under abiotic stress and improving plant protection against *Botrytis cinerea*, in contrast with the overexpressing IGs Arabidopsis line, where root colonization and beneficial effects of these endophytes were reduced (Poveda, 2021).

Indeed, the ability of fungal endophytes to deal with Brassicaceae GSLs can be a key factor in their colonization and interaction ability (Hiruma et al., 2018; Poveda et al., 2020b). Some endophytic fungi can colonize Brassicaceae roots by directly degrading GSLs, and especially sinigrin, as it has been described for *Fusarium oxysporum*, *Setophoma terrestris*, *Macrophomina phaseolina*, and *Paraphoma radicina* in horseradish (Szűcs et al., 2018). This may be due to the presence of myrosinase enzymes in these fungi, as it has been described in several Brassicaceae-associated rhizosphere and endophytic fungi (Ishimoto et al., 2000; Abdel-Fatah et al., 2021). The molecules derived from the enzymatic decomposition of the host’s GLSs (glucose and ITCs) can be used as nutrients by those fungi, or to gain a competitive advantage over less tolerant species (Szűcs et al., 2018). Thus, GSL and ITC root exudates may shape microbial rhizosphere composition and even be a potent allelopathic plant weapon via effects on mycorrhizal fungi (Cipollini et al., 2012). Even, myrosinase-producing fungi can act as plant allies by hydrolyzing GSLs and increasing the amount of toxic ITCs released to the rhizosphere (Ishimoto et al., 2004). A key question is how tolerant fungi deal with these toxic compounds, and several mechanisms have been described, mainly among fungal pathogens adapted to Brassicaceae (Plaszkó et al., 2021). One important mechanism relies on ITCs detoxification by fungal glutathione *S*-transferases (GSTs). Genome-mining of *Alternaria brassicicola* found 23 GST sequences (Calmes et al., 2015). Some of these GSTs were induced during *in planta* interaction and required for pathogenesis *in planta*, accepted allyl ITC as a substrate, and its inactivation resulted in ITC hypersensitivity. Instead of GSL hydrolysis and tolerance, another mechanism to overcome this defensive plant pathway is inhibiting it. *Thkel1* gene increases the root colonization ability of *T. harzianum* and reduces myrosinase activity of Arabidopsis and rapeseed plants (Poveda et al., 2019a). This gene encodes a protein with similarity to plant nitrile-specifier proteins (NSPs) and epithiospecifier proteins (ESPs) (Hermosa et al., 2011), which modulate myrosinases activity, resulting in GLSs degradation to nitriles and ETSs that is less toxic than ITCs (Martinez-Ballesta and Carvajal, 2015).

Apart from myrosinases and *T. harzianum* THKEL1 protein, fungal endophytes may make use of a diverse set of molecules to evade host defenses and colonize the plant. *Serendipita indica* encodes the fungal-specific β-glucan-binding lectin FGB1 that binds β-glucan with high specificity. This molecule alters fungal cell wall composition and properties and suppresses the immunity triggered upon β-glucan recognition by different plant hosts, such as Arabidopsis, barley, and *Nicotiana benthamiana* (Wawra et al., 2016). Proteins with carbohydrate-binding modules (CBMs) like FGB1 are enriched in *S. indica*, as well as in the genomes of other endophytic fungi, such as *Harpophora oryzae* and *C. tofieldiae* (Zuccaro et al., 2011; Xu et al., 2015; Hacquard et al., 2016). Specifically, proteins with chitin-binding CBM18 and LysM-chitin-binding domains CBM50, which are known as suppressors of host immunity against fungal plant pathogens by masking the MAMP chitin (de Jonge and Thomma, 2009; Lahrmann and Zuccaro, 2012; Romero-Contreras et al., 2019), and proteins with cellulose-binding CBM1 (Carbohydrate-Binding Module Family 1) modules, which are potentially involved in loosening the plant cell wall (Gaulin et al., 2002; Saloheimo et al., 2002). The expansion of these proteins compared with fungi with other lifestyles has been proposed as a genomic signature common to independently evolving root-associated fungal endophytes (Fesel and Zuccaro, 2016). Mesny et al. (2021) compared the genomes of 120 fungal isolates with different lifestyles including isolates representative of the Arabidopsis root mycobiota, identifying a set of 84 gene families associated with endophytism. Plant cell wall-degrading enzymes (PCWDEs), acting on xylan, cellulose, pectin, and hemicellulose were highly represented within these families, but also carbohydrate membrane transporters and genes involved in carbohydrate and amino acid metabolism (Mesny et al., 2021). Fungal endophytes also code a diverse repertoire of putative effector small secreted proteins (SSPs) (Mesny et al., 2021), although a reduction of effector repertoires and/or effector expression is observed when comparing the genomes of endophytes with closely related pathogens (Hacquard et al., 2016).

Besides the endophyte’s ability to evade or suppress the plant immune system, it is the plant itself that could finally allow being colonized, depending on the benefit expected from it (Thoms et al., 2021). A link between plant nutrition and immunity status has been hypothesized (Walters and Bingham, 2007; Wang et al., 2011; Dindas et al., 2022) with mechanisms that could modulate the plant immune system to accommodate the entrance and plant colonization of beneficial microorganisms under nutrients scarcity (Zipfel and Oldroyd, 2017; Shi et al., 2021; Thoms et al., 2021; Tang et al., 2022). Low available phosphate (Pi) in the soil enhances AMF colonization of host plants, whereas this is decreased under high Pi fertilization, although it is still unclear if immunity modulation by the plant is directly involved (Karandashov and Bucher, 2005; Nouri et al., 2014; Kobae et al., 2016). The possible link between plant immunity, Pi deficiency, and plant colonization seems more clear in the system formed by the fungal endophyte *C. tofieldiae*, which enhances Pi uptake and promotes plant growth, and Arabidopsis (Hiruma et al., 2016; Frerigmann et al., 2020). In this case, Pi deficiency increases colonization of Arabidopsis by *C. tofieldiae*, and transcriptomic analyses showed that defense-related responses were significantly reduced during *C. tofieldiae* root colonization in low Pi conditions compared to optimal conditions (Hacquard et al., 2016; Hiruma et al., 2016; Frerigmann et al., 2020). In contrast, low Pi conditions did not alter the plant immune response against colonization by *Colletotrichum incanum*, a close relative of *Colletotrichum tofeldiae* that behaves as a pathogen and is a less Pi supplier. On the other side, plant nutrient status did not significantly alter the expression of *C. tofeldiae* candidate effector genes. All this evidence suggests that, in this system, it is the plant, and not the fungus, who mainly controls the outcome of the interaction through the modulation of its defensive response in reaction, in turn, to fungal-mediated Pi transfer (Hacquard et al., 2016; Hiruma et al., 2016). Hiruma et al. (2016) also found a link between this defense response modulation and the phosphate starvation response (PSR) system through MYB transcription factors, PHR1 and PHL1, which positively regulate root colonization of *C. tofieldiae*. PHR1 and PHL1 control most transcriptional activation and repression responses to phosphate starvation (Bustos et al., 2010). An important part of the genes regulated by PHR is known or predicted to mediate biosynthesis of defense-related IG (Castrillo et al., 2017). This fact, together with the need for an intact PEN2-myrosinase-dependent IG pathway for the beneficial interaction with *C. tofieldiae*, points, again, to the relevance of these compounds in regulating Brassicaceae interactions with fungal endophytes (Hiruma et al., 2016; Frerigmann et al., 2020; Zimmermann et al., 2021).

## Benefits of fungal endophytes to Brassicaceae: From plant models to crops

The ability of endophytic fungi to provide benefits to the plant is widely reviewed nowadays, highlighting their importance in the development of sustainable agriculture (Carla et al., 2021; Poveda et al., 2021). There are many beneficial effects described in crop plants, which include the following: plant growth promotion (PGP), yield increase, crop quality improvement, and enhanced tolerance to abiotic and biotic stresses (Supplementary Table 1). Despite the growing evidence of their potential applications in agriculture, the molecular mechanisms underlying these positive interactions have not been elucidated in many cases. The growing number of reports about the impact of endophytic fungi on growth and physiological parameters are not usually accompanied by deeper analyses that can explain the mechanisms by which they manipulate plant physiology and exert their effects. For example, root colonization of cabbage, rapeseed, black mustard, cress, hoary stock or flixweed by *Serendipita vermifera*, or rapeseed by *Alternaria alternata* or *Leptosphaeria biglobosa*, have been reported to significantly increase root and stem biomass (Dolatabadi and Goltapeh, 2013; Zhang Q. et al., 2014), but without describing the mechanism of action behind this effect. This is likely due to the lack of genomic information and mutant lines for crop plant species and the difficulty of establishing experiments under highly controlled conditions (*in vitro* or growth chambers) (Koornneef and Meinke, 2010). For these reasons, Arabidopsis has also been used as a good model to understand how endophytic fungi interact with the plant.

### Plant growth promotion and increase in yield and quality

Beneficial fungi usually display different effects on plants that ultimately result in PGP (Poveda et al., 2021; Sarkar et al., 2021). The fungal endophyte may have different modes of action that could be simultaneously contributing to the benefits obtained by the plant (Yan et al., 2019; Sarkar et al., 2021). These mechanisms include the following: the facilitation of access to nutrients and enhanced nutrient uptake, an increase of photosynthetic rates, and regulation of the action of secondary metabolites, such as phytohormones or others with undescribed functions (Poveda et al., 2021).

Nutrient supply via nutrient uptake is one of the main beneficial functions attributed to fungal endophytes (Poveda et al., 2021; Sarkar et al., 2021). This benefit from fungal endophytes takes more relevance in Brassicaceae plants, which cannot establish functional mycorrhizal symbiotic associations (Cosme et al., 2018; Hiruma et al., 2018; Sarkar et al., 2021). Fungal endophytes can increase the plant content of all macronutrients and many micronutrients (Behie and Bidochka, 2014; García-Latorre et al., 2021). The mechanisms are not clear in most cases, but they can broadly be divided into three groups: (i) the increasing availability of soil nutrients (such as metabolization of macromolecules, mineralization and solubilization of organic or insoluble forms, or chelation of cations that can therefore be better assimilated by the plant), (ii) the transfer of nutrients from the soil to the root, acting as root extensions that transport nutrients to the plant, and (iii) the induction of changes in the plant, such as the promotion of root growth and development, which increases its absorption ability, or promoting the activity of plant enzymes or transporters (Behie and Bidochka, 2014; García-Latorre et al., 2021; Sarkar et al., 2021).

Phosphorus (P) is one of the three essential macronutrients for plant development (Maathuis, 2009) and, hence, the tripartite interplay among P, endophytes, and plants has been deeply studied (Behie and Bidochka, 2014; Hiruma et al., 2018; Mehta et al., 2019; Bhalla et al., 2022). The fungal endophyte *C. tofieldiae* has been embraced as a fungal model to analyze endophyte-plant associations related to efficient inorganic phosphorus (Pi) utilization in non-mycorrhizal plants, such as *A. thaliana* (Fesel and Zuccaro, 2016; Hiruma et al., 2018). This endophyte was isolated from a wild population of *A. thaliana* growing in poor-nutrient soils (García et al., 2013), and established a mutualistic interaction with *A. thaliana*, promoting plant growth and fertility under Pi starvation conditions (Hiruma et al., 2016). The observed PGP phenotype and the increased tolerance to Pi-limiting conditions seem to be mainly the result of two combined mechanisms: Pi translocation to plant tissues, and the upregulation of the Arabidopsis genes AtPHT1;2 and AtPHT1;3 related to Pi transport. In the interacting fungus, in turn, the two most highly upregulated genes under low Pi conditions were an acid phosphatase and a phosphate H^+^ symporter, which can be related to Pi solubilization and uptake, respectively (Hiruma et al., 2016). Besides Arabidopsis, *C. tofieldiae* colonizes and promotes the growth of other two very different hosts: tomato and maize (Díaz-González et al., 2020). However, in these hosts, *C. tofieldiae* promotes growth and yield under optimal P fertilization. These results point at additional unknown mechanisms involved in PGP that may change depending on the host and be modulated by the plant growth conditions.

The transformation of non-bioavailable sources of nutrients into inorganic forms, which can be assimilated by plants, has also been pointed out as a PGP mechanism. Solubilization of P is a well-extended trait among these microorganisms. Some examples of P-solubilizing endophytic fungi studied in Brassicaceae crops roots are *Alternaria* spp., *Fusarium* spp., *Mucor* spp., or *Penicillium* spp. (Shi et al., 2017; Zahoor et al., 2017). Fungal endophytes can also induce phosphatase activity in colonized roots, increasing acid release in root exudates and P availability to the plant, as reported in rapeseed roots colonized by *S. indica* (Wu et al., 2018). The transformation of organic nitrogen into bioavailable forms mediated by endophytic fungi has also been proposed (Usuki and Narisawa, 2017; Khastini and Jannah, 2021). Two different studies on Chinese cabbage (*B. rapa*) suggested that the inoculation with different endophytic fungi enabled the use of the amino acids Gly, Val, Leu, and Phe as sources of nitrogen for the plant (Usuki and Narisawa, 2017; Khastini and Jannah, 2021). Although further evidence supporting this hypothesis is needed, the studies indicated that the endophytic fungi could transform the amino acids into nitrogen forms, which can be effectively metabolized by the plant, as well as facilitate nitrogen uptake through fungal hyphae.

Along with the increased nutrient supply, endophytic fungi can promote plant growth through the modulation of photosynthesis rates. Studies of this effect on Brassicaceae plants are few and are mainly focused on Arabidopsis. One example is the work of Rozpądek et al. (2019), which showed that the fungal endophyte *Mucor* sp. optimized photosynthesis and carbon uptake through the upregulation of genes involved in typical photosynthesis response to carbon deficiency. This resulted in an improved plant CO_2_ assimilation and water use efficiency that finally led to increased growth of Arabidopsis plants. Similarly, *Trichoderma* is known to regulate the expression of genes related to photosynthetic systems and sugar metabolism (Shoresh et al., 2010; Harman et al., 2021). A recent study demonstrated that *T. atroviride* can modulate the expression of the sucrose transporter AtSUC2 (Esparza-Reynoso et al., 2021). The overexpression of this gene mediated by fungal volatiles resulted in sucrose-enriched root exudates and increased sugar supply to the shoots and roots, thus, improving Arabidopsis development and root branching.

Many fungi regulate plant growth by the production of secondary metabolites or the modulation of phytohormone signaling and related gene expression in the plant (Sharma et al., 2021). The production of indole-acetic acid (IAA) and other auxins and the influence on auxin metabolism has a direct effect on root architecture (Aloni et al., 2006). Recent studies with different endophytic fungi co-cultivated with Arabidopsis indicate that the molecular cross-talk between the plant and the endophyte impacts the biosynthesis of auxins in both partners, leading to the stimulation of lateral root formation (Liao et al., 2017; Inaji et al., 2020; Sun et al., 2020). This effect on root development might finally result in an overall plant growth promotion. One example was reported by Contreras-Cornejo et al. (2009), in which *Trichoderma virens* increased lateral root and plant growth in Arabidopsis through auxin-dependent mechanisms. Further evidence is found in the inoculation of Chinese cabbage with *S. indica*, in which the upregulation of auxin signaling and transport genes, such as *AUX1*, led to enhanced root hair development and increased seedling growth (Lee et al., 2011). Additional examples of IAA-producing fungi among Brassicaceae crops are *Alternaria* spp., *Fusarium* spp., *Mucor* spp., or *Penicillium* spp. (Shi et al., 2017; Zahoor et al., 2017).

Besides auxins, the synthesis and manipulation of other phytohormones have been reported. Several studies point out the ability of fungal endophytes to synthesize and induce responses to cytokinin as key factors for their beneficial effect (Vadassery et al., 2008; Bean et al., 2021). As an example, Vadassery et al. (2008) demonstrated that *trans*-Zeatin and the CRE1/AHK2 receptor played critical roles in the promotion of Arabidopsis plant growth by *S. indica*. In addition, the plant-growth-inhibiting effect of ethylene can be counteracted by the synthesis of the enzyme 1-aminocyclopropane-1-carboxylate (ACC) deaminase (Singh et al., 2015). This enzyme, which is commonly secreted by PGP bacteria (Glick, 2005; Glick et al., 2007), binds to ACC, lowering the levels of ethylene in the plant. The biosynthesis of ACC deaminase has also been described in fungi (Nascimento et al., 2014). For instance, it seems to play a relevant role in the promotion of root elongation driven by *Trichoderma asperellum* in canola roots since mutant strains defective in the ACCD gene showed no ability to improve root development (Viterbo et al., 2010). The production of gibberellins (GAs) has also a direct relationship with plant vegetative and reproductive development (Gao and Chu, 2020). In Chinese cabbage roots, the endophytic fungus *Neosartorya* sp. can produce the gibberellic acids GA1, GA3, GA4, GA7, GA9, and GA15, thus, increasing plant length and biomass (Hamayun et al., 2011).

Apart from vegetative growth, PGP can also affect fruits and seed yield, either as a direct consequence of a better nutritional status of the plant, or by phytohormonal changes that promote the development of reproductive structures. For example, in rapeseed plants, root-colonization by *T. harzianum*, implied a significant increase in the number of siliques per plant (Poveda et al., 2019a,b). Several authors have reported the ability of fungal endophytes to regulate Arabidopsis flowering mechanisms. In two different studies, the fungal endophytes *Pochonia chlamydosporia* and *S. indica* accelerated floral transition and increased seed production by modulating the expression of key genes involved in flowering time, such as FLOWERING LOCUS T (FT), LEAFY (LF) or FLOWERING LOCUS C (Kim et al., 2017; Zavala-Gonzalez et al., 2017). In contrast to *P. chlamydosporia*, the effect of *S. indica* was also related to upregulation of gibberellin biosynthetic genes (Kim et al., 2017).

Besides increasing plant growth and yield, endophytic fungi can induce systemic changes in their host plant, increasing nutraceutical components in their tissues and improving the quality of derived foods. In different leafy *Brassica* vegetables, root colonization by *Trichoderma hamatum* increased the accumulation of GSLs and antioxidant activity, improving their nutritional quality (Velasco et al., 2021). Similarly, *S. indica* increased chlorophyll, carotenoids, and flavonoids accumulation in Chinese cabbage leaves (Khalid et al., 2017). On the other hand, these fungi can decrease the accumulation of secondary metabolites detrimental to health. This is the case of *S. indica* in rapeseed plants where, along with an increase in seed yield, the fungus decreases the expression in the seeds of genes related to the synthesis of erucic acid, a compound that is toxic to humans (Su et al., 2017). The mechanisms behind these changes could be related to the modulation by fungal endophytes of the production of secondary metabolites related to biotic or abiotic stresses (see below), although other non-described mechanisms could also be involved.

### Abiotic stress tolerance

The role of endophytic microorganisms in increasing plant tolerance to abiotic stresses has been extensively studied in recent years (Lata et al., 2018; Eid et al., 2019). Endophytic fungi can act directly on the stress-causing agent and reduce it, synthesize compounds that reduce plant stress or induce a specific response in the plant that increases tolerance to the stress (Lata et al., 2018; Eid et al., 2019).

One of the most damaging anthropogenic abiotic stresses for plants is the contamination of soil and water with heavy metals. To isolate endophytic fungi that promote plant tolerance or phytoremediation of heavy metals, existing populations in contaminated environments are often studied (Deng and Cao, 2017). This has also been carried out with different Brassicaceae grown in mines or other contaminated environments (Jiang et al., 2008; Diene et al., 2014; Liu et al., 2017), isolating endophytes, such as dark septate fungi or *Mucor* sp. CBRF59, that have shown to increase plant tolerance to Cd, Pb, Cr, Mn, Co, Cu, or Zn (Deng et al., 2011, 2013; Zahoor et al., 2017). The mechanisms involved in this effect include both organic degradation of heavy metal compounds and their accumulation in hyphae (Deng et al., 2011, 2013; Zahoor et al., 2017). Besides, endophytic fungi may make use of self-produced siderophores, which are molecules involved in iron chelation that can also show an affinity for different heavy metals. Thus, the production of siderophores by *Alternaria* sp., *Fusarium* sp., and *Penicillium* sp. has been related to increased tolerance and phytoextraction of Cd and Pb in rapeseed plants (Shi et al., 2017).

Under stress conditions, the enhanced protection against oxidative stress seems to be a common mechanism in mutualistic interactions between plants and fungal endophytes. The production of antioxidants, such as phenolic compounds (phenilpropanoids and flavonoids) or enzymes (i.e., catalase and ascorbate peroxidase) or their induction in the host, are the mechanisms involved in this effect (Hamilton et al., 2012). This mechanism has been reported in cauliflower plants inoculated with *T. harzianum* in Pb-contaminated soils (Afshari et al., 2018). Also, under drought and salinity, Chinese cabbage roots colonized by *S. indica* tolerated the stress due to the fungal induction of antioxidant activity in the host plant, together with the expression of drought/salinity response genes and an accumulation in leaves of specific thylakoid (CAS) proteins (Sun et al., 2010; Khalid et al., 2018). Similarly, Indian mustard and rapeseed plants root-colonized by *Trichoderma* responded to salinity and drought by increasing their antioxidant activity (Ahmad et al., 2015).

Phytohormones also play crucial roles under salt and water stress. IAA seems to be involved in *Trichoderma*-mediated tolerance of Arabidopsis to saline conditions (Contreras-Cornejo et al., 2014). As a result, Na^+^ excretion through root exudates might have been facilitated by the increased number of lateral roots and root hairs. Additionally, inoculated plants showed higher levels of abscisic acid (ABA), L-proline, and ascorbic acid. Likewise, the inoculation of *B. napus* with *Trichoderma parareesei* induced the expression of stress tolerance genes related to ABA under water stress, and ethylene under salt stress (Poveda, 2020).

Fungal-mediated plant thermotolerance may be due to particular mechanisms, although it has not been much investigated in Brassicaceae. However, some studies have revealed promising results in Arabidopsis, both under heat and cold stress. For example, González-Pérez et al. (2018) showed that volatiles produced by *T. virens* and *T. atroviride* positively impacted the growth of Arabidopsis seedlings subjected to low temperatures. Moreover, plants exposed to *Trichoderma* volatiles showed overexpression of the *ERD14* gene, related to protection against dehydration and involved in the response to different abiotic stresses, including cold (Kovacs et al., 2008; González-Pérez et al., 2018). In another study, the co-cultivation of Arabidopsis plants with the fungus *Paraphaeosphaeria quadriseptata* increased plant heat stress tolerance (McLellan et al., 2007). The research suggested that the acquired thermotolerance was due to the fungal production of monicillin I (MON), which inhibits HEAT SHOCK PROTEIN90 (HSP90), thereby, inducing the activation of heat stress transcription factors (HSF) and the transcription of HSF-dependent components of the plant heat shock response, such as HSP101 and HSP70 (McLellan et al., 2007).

### Biotic stress tolerance: Endophytic fungi as biological control agents and inducers of systemic resistance

Numerous isolates of endophytic fungi have been identified as efficient biological control agents (BCA) against plant pathogens and pests. This way, fungal endophytes, together with other microorganisms, pose a sustainable alternative to chemical pesticides (Mantzoukas and Eliopoulos, 2020; Fontana et al., 2021). One mechanism of action for plant protection by fungal endophytes is the priming of plant defense reactions through the elicitation of local or systemic responses after the recognition of the fungus by the plant, as explained in previous sections (Fadiji and Babalola, 2020; Sarkar et al., 2021). However, endophytic fungi can also act directly by competition, antagonism, or parasitism, including the production of antimicrobial compounds (De Silva et al., 2019; Fadiji and Babalola, 2020).

In Chinese cabbage and kale plants, it has been reported that a significant reduction of leaf lesions caused by *Pseudomonas syringae* pv. *maculicola* and *Xanthomonas campestris* pv. *campestris* and the fungal pathogen *A. alternata* was produced by systemic activation of plant defenses by different root endophytic fungi, such as *Heteroconium chaetospira*, *Acrocalymma vagum*, *Curvularia* sp., *Phialocephala* sp., *S. terrestris*, or *T. hamatum* (Morita et al., 2003; Poveda et al., 2020c). *S. indica* acts against the oomycete *Plasmodiophora brassicae*, the causal agent of clubroot disease, by inducing local defensive responses in the roots of Chinese cabbage, increasing the local flavonoid content, and reducing gall formation by the pathogen up to 60% (Khalid et al., 2020). However, *S. indica* was not effective in controlling clubroot in rapeseed plants, highlighting the fungus-plant specificity in the effectiveness of biological control strategies (Sedaghatkish et al., 2021). *H. chaetospira* also protects plants against clubroot disease, in this case by space competition, with reductions in disease incidence up to 100% (Narisawa et al., 1998, 2004, 2005). The pathogen *Sclerotinia sclerotiorum* behaves as an endophyte when infected with the small-DNA mycovirus SsHADV-1, activating the root defenses of the host plant, reducing the disease severity of rapeseed stem rot and improving yield (Zhang et al., 2020). Thus, different endophytic fungi can show different mechanisms of action against the same plant pathogen. Even the plant can selectively modify the composition of endophytes upon infection, favoring the presence of efficient BCAs against the pathogen, as also described for combating *P. brassicae* (Zhao et al., 2017; Lebreton et al., 2019; Tian et al., 2019).

Mycoparasitism, competition for space and nutrients, and the production of antifungal compounds have been reported to be very effective in inhibiting the growth of fungal pathogens, such as *B. cinerea*, *S. sclerotiorum*, or *Sclerotinia trifoliorum* (Zhang Q. et al., 2014; Qin et al., 2019). A variety of antagonistic species against fungi has been isolated from different organs of rapeseed plants, such as *Aspergillus capensis*, *Chaetomium globosum*, *Clonostachys rosea*, *L. biglobosa*, or *Simplicillium lamellicola*, among many others (Zhang Q. et al., 2014; Qin et al., 2019). Furthermore, endophytic fungi of Brassicaceae crops can develop combined mechanisms of action as BCAs of pathogenic fungi. In this sense, root colonization of Chinese cabbage by *Veronaeopsis simplex* resulted in a reduction of more than 70% in *Fusarium* wilt disease. This effect was due to several mechanisms of action, including competition for rhizosphere and root space, production of siderophores that reduced Fe availability to the pathogen, activation of host plant-root defenses, and an increased diversity and number of possibly antagonistic fungi in the rhizosphere (Khastini et al., 2012).

So far, many species of endophytic fungi have been described with the ability to act directly and indirectly against plant-parasitic nematodes (Poveda et al., 2020a). As far as Brassicaceae crops are concerned, there are only a few publications on the use of endophytic fungi as BCAs against these plant parasites. One example is *T. harzianum*, which reduces the disease caused by *Meloidogyne incognita* in turnip plants, although the mechanism of action involved is unknown (Ibrahim et al., 2012). In wild turnip plants, root colonization by the endophyte *F. oxysporum* caused a slowdown in the development of the root nematode cysts, *Heterodera schachtii*, due to the volatiles emitted by the fungus (Moisan et al., 2021).

Some endophytic fungi of Brassicaceae crops can act directly on insect-pest larvae by parasitism, as they are present in the plant tissues consumed by them. Thus, consumption of cauliflower roots colonized by *Trichoderma koningiopsis* caused larval death of the fly *D. radicum* (Razinger et al., 2018). The same happened with larvae of the lepidopteran *P. xylostella* fed with rapeseed leaves colonized by *Metarhizium anisopliae* (Batta, 2013). On the other hand, endophytic fungi of Brassicaceae crops can also activate the systemic resistance of their host plant against herbivores by colonizing the roots. For example, in Brussels sprouts and cabbage plants root-colonized by *Acremonium alternatum*, a systemic accumulation of phytosterol reduced feeding by *P. xylostella* larvae, increasing their mortality (Raps and Vidal, 1998).

Furthermore, endophytic fungi from Brassicaceae crops can be used as biotechnological tools for genetic engineering, improving their ability to control agricultural pests. The fungus *C. globosum*, isolated from rapeseed plants, was transformed with the agglutinin gene from the plant *Pinellia ternata*. Rapeseed plants colonized by this transgenic endophytic fungus significantly inhibited the growth and reproduction of aphid *Myzus persicae*, due to the accumulation of agglutinin (Qi et al., 2011). In general, the use of endophytic fungi in the integrated pest management of Brassicaceae crops can be an efficient strategy. However, it is important to previously study the effect of the use of insecticides on the endophytic fungal microbiota of these plants, since it has been shown that it is significantly affected (Zhou et al., 2016).

## Conclusion

The Brassicaceae is a noteworthy plant family. It includes important crops, namely, the genus *Brassica* (Jabeen, 2020; Raza et al., 2020), and the essential model plant *A. thaliana* (Koornneef and Meinke, 2010). Members of the family present remarkable particularities, such as the presence of a diverse number of defenses related to secondary metabolites, mainly in the group of GSLs (Halkier and Gershenzon, 2006), and the inability to form mutualistic associations with mycorrhizal fungi (Cosme et al., 2018). In this context, the beneficial interactions of this plant family with endophytic fungi acquire special relevance. Endophytic fungi may provide Brassicaceae plants with the benefits that mycorrhiza cannot offer. For that, fungal endophytes must adapt to the GLS based particular immune system of Brassicaceae and may have co-opted conserved mechanisms of interactions with mycorrhiza (Hiruma et al., 2018; Poveda et al., 2020b).

*A. thaliana* is one of the most famous Brassicaceae species. This model plant has provided us with a great part of the knowledge of plant genetics and molecular biology (Koornneef and Meinke, 2010). More recently, the study of its wild populations and other related wild Brassicaceae is also bringing much understanding about plant ecology (Bergelson and Roux, 2010). Thus, much of the knowledge on the interactions of plants with microorganisms comes from the Arabidopsis model (Jones and Dangl, 2006; Bacete et al., 2018; Ngou et al., 2022). This knowledge has been greatly biased toward pathogenic interactions, probably because Arabidopsis cannot be used as a model for the best-known mutualistic interactions with microorganisms, such as nodulating rhizobia or mycorrhizal fungi (Thoms et al., 2021). Lately, the study of alternative beneficial interactions, such as those established with fungal endophytes, is experiencing a significant expansion, and the development of novel models of study with fungal endophytes that interact with Arabidopsis is greatly contributing to it (Fesel and Zuccaro, 2016; Hiruma et al., 2018). These novel models bring us the opportunity to study both the particularities involved in the interactions between these microorganisms and the Brassicaceae plants and the subjacent generalities that can be extended to other plant species, including conserved features with the lost mycorrhization abilities. Thus far, most of the questions about the interactions of fungal endophytes with plants in general, and with Brassicaceae in particular, are open. The extension and application of this knowledge to Brassicaceae and other crops is still a pending subject that should be approached.

The high diversity and the particularities of the Brassicaceae family point to these plants as unique sources for the discovery of novel beneficial interactions with fungal endophytes that can be applied for a more efficient and sustainable agricultural production, not only to Brassicaceae crops but also to other agricultural species (Díaz-González et al., 2020; Poveda et al., 2020c). Let’s take advantage of it.

## Author contributions

SS proposed the review structure and content and coordinated the work of all authors. JP, SD-G, MD-U, PV, and SS wrote sections of the manuscript. JP, MD-U, and PV prepared the tables and figures. SD-G compiled all sections and wrote the first draft. All authors contributed to manuscript revision, read, and approved the submitted version.
