# Supplementary material for: Fungal endophytes of Brassicaceae: Molecular interactions and crop benefits
Source: Front Plant Sci. 2022 Aug 5;13:932288. doi: 10.3389/fpls.2022.932288 (PMC9390090; doi:10.3389/fpls.2022.932288)
Supplement: Supplementary file 1 [file Table_1.pdf]

1 Supplementary Table

Table 1. Compilation of studies on filamentous endophytic fungi in Brassicaceae crops, indicating the effects on host plants, the mechanisms of action involved, and the way in which the fungi colonize plant tissues.

ENDOPHYTIC FUNGUS	BRASSICACEAE CROP	EFFECT	MECHANISM OF ACTION	TISSUE COLONIZATION/ MODE	REFERENCES
Acremonium alternatum	Brussels sprouts (*Brassica oleracea* var. *gemmifera*)	*Plutella xylostella* larvae mortality	Systemic defenses activation	Roots / no mechanism identified	Dugassa-Gobena et al. (1998)
	Cabbage (*B. oleracea* var. *capitata*)	*P. xylostella* larvae mortality	Systemic defenses activation	Roots / no mechanism identified	Raps and Vidal (1998)
	Rapeseed (*Brassica napus*)	Increased disease resistance against protista *Plasmodiophora brassicae*	Unidentified	Roots / no mechanism identified	Auer and Ludwig-Müller (2014)
Acrocalymma vagum	Kale (*B. oleracea* var. *acephala*)	Plant growth promotion<br>Plant tolerance under cold stress<br>Increased disease resistance against bacteria *Xanthomonas campestris* pv. *campestris*	Systemic defenses activation	Roots / no mechanism identified	Poveda et al. (2020)
Alternaria alternata	Rapeseed (*B. napus*)	Plant growth promotion	Unidentified	Roots, stems and leaves/ no mechanism identified	Zhang et al. (2014)
Alternaria sp.	Rapeseed (*B. napus*)	Antagonism against fungus *S. sclerotiorum*	Mycoparasitism or competition for space and nutrients	Stems/ no mechanism identified	Zhang et al. (2014)
	Rapeseed (*B. napus*)	Increased phytoextraction and plant-tolerance to Cd and Pb<br>Plant growth promotion	Siderophores production<br>IAA production<br>P solubilization	Roots / no mechanism identified	Shi et al. (2017)
Aspergillus capensis	Rapeseed (*B. napus*)	*In vitro* growth inhibition of fungi *Botrytis cinerea*, *Monilinia fructicola*, *Sclerotinia sclerotiorum* and *S. trifoliorum*	Non-volatile antifungal compounds production	Unidentified	Qin et al. (2019)
Aspergillus flavipes	Rapeseed (*B. napus*)	Antagonism against fungus *S. sclerotiorum*	Non-volatile antifungal compounds production	Stems/ no mechanism identified	Zhang et al. (2014)
Aspergillus sp.	Indian mustard (*Brassica juncea*)	Increased phytoextraction and plant-tolerance to Cd and Ni	Unidentified	Roots/ no mechanism identified	Jiang et al. (2008)
Basipetospora sp.	Pakchoi or Chinese cabbage (*Brassica campestris* var. *chinensis/pekinensis*; *B. rapa* var. *chinensis/pekinensis*)	Plant growth promotion	N supply<br>Auxins production	Roots/ no mechanism identified	Khastini and Jannah (2021)
Beuveria bassiana	Rapeseed (*B. napus*)<br>Cabbage (*B. oleracea* var. *capitata*)<br>Turnip (*Brassica rapa*)	Reduced disease caused by fungus *Leptosphaeria maculans*	Unidentified	Roots and stem/ no mechanism identified	Roodi et al. (2021)
Chaetomium globosum	Rapeseed (*B. napus*)	Antagonism against fungus *S. sclerotiorum*	Non-volatile antifungal compounds production	Roots/ no mechanism identified	Zhang et al. (2014)
Chaetomium sp.	Kale (*B. oleracea* var. *acephala*)	Plant tolerance under cold stress	Unidentified	Roots / no mechanism identified	Poveda et al. (2020)

ENDOPHYTIC FUNGUS	BRASSICACEAE CROP	EFFECT	MECHANISM OF ACTION	TISSUE COLONIZATION/ MODE	REFERENCES
Cladosporium sp.	Cauliflower (*Brassica oleracea* var. *botrytis*)	Increased resistance to *Spodoptera litura* damage	Systemic defenses activation	Roots / no mechanism identified	Thakur et al. (2013)
Clonostachys rosae	Rapeseed (*B. napus*)	Antagonism against fungus *S. sclerotiorum*	Non-volatile antifungal compounds production	Roots and stems/ no mechanism identified	Zhang et al. (2014
Curvularia sp.	Kale (*B. oleracea* var. *acephala*)	Plant growth promotion<br>Increased disease resistance against bacteria *X. campestris* pv. *campestris*	Systemic defenses activation	Roots / no mechanism identified	Poveda et al. (2020)
Diaporthe sp.	Kale (*B. oleracea* var. *acephala*)	Plant tolerance under cold stress	Unidentified	Roots / no mechanism identified	Poveda et al. (2020)
Dothidea sp.	Rapeseed (*B. napus*)	Antagonism against fungus *S. sclerotiorum*	Mycoparasitism or competition for space and nutrients	Stems/ no mechanism identified	Zhang et al. (2014)
Epicocum nigrum	Rapeseed (*B. napus*)	Antagonism against fungus *S. sclerotiorum*	Mycoparasitism or competition for space and nutrients	Stems and leaves/ no mechanism identified	Zhang et al. (2014)
Fusarium clamydosporium	Rapeseed (*B. napus*)	Antagonism against fungus *S. sclerotiorum*	Mycoparasitism or competition for space and nutrients	Stems/ no mechanism identified	Zhang et al. (2014)
F. oxysporum	Rapeseed (*B. napus*)	Antagonism against fungus *S. sclerotiorum*	Volatile antifungal compounds production	Roots and stems/ no mechanism identified	Zhang et al. (2014)
	Horseradish (*Armoracia rusticana*)	-	-	Roots/ GSLs degradation by fungus	Szűcs et al. (2018)
	Kale (*B. oleracea* var. *acephala*)	Plant growth promotion<br>Plant tolerance under cold stress<br>Increased disease resistance against bacteria *X. campestris* pv. *campestris*<br>Increased resistance to *Mamestra brassicae* damage	Systemic defenses activation	Roots / no mechanism identified	Poveda et al. (2020)
	Wild turnip (*B. rapa* var. *silvestris*)	Increased disease resistance against nematode *Heterodera schachtii*	Fungal volatiles slowed down the development of the root nematode cysts	Roots/ no mechanism identified	Moisan et al. (2021)
F. proliferatum	Rapeseed (*B. napus*)	Antagonism against fungus *S. sclerotiorum*	Mycoparasitism or competition for space and nutrients	Roots and leaves/ no mechanism identified	Zhang et al. (2014)
F. solani	Rapeseed (*B. napus*)	Antagonism against fungus *S. sclerotiorum*	Mycoparasitism or competition for space and nutrients	Roots / no mechanism identified	Zhang et al. (2014)
F. tricinctum	Rapeseed (*B. napus*)	Antagonism against fungus *S. sclerotiorum*<br>Plant growth promotion	Mycoparasitism or competition for space and nutrients	Roots / no mechanism identified	Zhang et al. (2014)
Fusarium sp.	Rapeseed (*B. napus*)	Increased phytoextraction and plant-tolerance to Cd and Pb<br>Plant growth promotion	Siderophores production<br>IAA production<br>P solubilization	Roots / no mechanism identified	Shi et al. (2017)
Helminthosporium velutinum	Pakchoi or Chinese cabbage (*B. campestris* var.	Increased phytoextraction and plant-tolerance to Cs	Unidentified	Roots / no mechanism identified	Diene et al. (2014)

ENDOPHYTIC FUNGUS	BRASSICACEAE CROP	EFFECT	MECHANISM OF ACTION	TISSUE COLONIZATION/ MODE	REFERENCES
	chinensis/pekinensis; *B. rapa* var. *chinensis/pekinensis*)				
Heteroconium chaetospira	Pakchoi or Chinese cabbage (*B. campestris* var. *chinensis/pekinensis*; *B. rapa* var. *chinensis/pekinensis*)	Increased disease resistance against protista *P. brassicae* and fungus *Verticillium dahliae*	Unidentified	Roots / no mechanism identified	Narisawa et al. (2000)
	Pakchoi or Chinese cabbage (*B. campestris* var. *chinensis/pekinensis*; *B. rapa* var. *chinensis/pekinensis*)	Increased disease resistance against bacteria *Pseudomonas syringae* pv. *maculicola* and fungos *Alternaria brassicae*	Systemic defenses activation	Roots / no mechanism identified	Morita et al. (2003)
	Pakchoi or Chinese cabbage (*B. campestris* var. *chinensis/pekinensis*; *B. rapa* var. *chinensis/pekinensis*)	Increased disease resistance against protista *P. brassicae*	Competition for space	Roots / no mechanism identified	Narisawa et al. (1998)
	Pakchoi or Chinese cabbage (*B. campestris* var. *chinensis/pekinensis*; *B. rapa* var. *chinensis/pekinensis*)	Increased disease resistance against protista *P. brassicae*	Unidentified	Roots / no mechanism identified	Narisawa et al. (2004)
	Pakchoi or Chinese cabbage (*B. campestris* var. *chinensis/pekinensis*; *B. rapa* var. *chinensis/pekinensis*)	Increased disease resistance against protista *P. brassicae*	Unidentified	Roots / no mechanism identified	Narisawa et al. (2005)
	Pakchoi or Chinese cabbage (*B. campestris* var. *chinensis/pekinensis*; *B. rapa* var. *chinensis/pekinensis*)	Plant growth promotion	N supply	Roots / no mechanism identified	Usuki and Narisawa (2017)
Humicola sp.	Pakchoi or Chinese cabbage (*B. campestris* var. *chinensis/pekinensis*; *B. rapa* var. *chinensis/pekinensis*)	Plant growth promotion	N supply<br>Auxins production	Roots / no mechanism identified	Khastini and Jannah (2021)
Leptosphaeria biglobosa	Rapeseed (*B. napus*)	Antagonism against fungus *S. sclerotiorum*<br>Plant growth promotion	Non-volatile antifungal compounds production	Roots and stems/ no mechanism identified	Zhang et al. (2014)
Macrophomina phaseolina	Horseradish (*A. rusticana*)	-	-	Roots/ GSLs degradation by fungus	Szűcs et al. (2018)
Meliniomyces variabilis	Pakchoi or Chinese cabbage (*B. campestris* var. *chinensis/pekinensis*; *B. rapa* var. *chinensis/pekinensis*)	Increased disease resistance against protista *P. brassicae*	Competition for space	Roots / no mechanism identified	Narisawa et al. (2004)
Metarhizium anisopliae	Rapeseed (*B. napus*)	*P. xylostella* larvae mortality	Direct parasitism by larvae feed in fungus-colonized tissues	Leaves colonization/ no mechanism identified	Batta (2013)
Muscodor albus	Kale (*B. oleracea* var. *acephala*)	Antagonism against oomycete *Pythium ultimum*	Volatile antifungal compounds production	Roots / no mechanism identified	Worapong and Strobel (2009)
Mucor sp.	Pakchoi or Chinese cabbage (*B. campestris* var. *chinensis/pekinensis*; *B. rapa* var. *chinensis/pekinensis*)	Increased plant-tolerance to Cd and Pb	Hyphae-accumulation of heavy metals	Roots / no mechanism identified	Deng et al. (2011)

ENDOPHYTIC FUNGUS	BRASSICACEAE CROP	EFFECT	MECHANISM OF ACTION	TISSUE COLONIZATION/ MODE	REFERENCES
	Rapeseed (*B. napus*)	Increased phytoextraction and plant-tolerance to Cd and Pb	Organic degradation of heavy metals	Roots / no mechanism identified	Deng et al. (2013)
	Field mustard (*Brassica campestris*; *B. rapa* var. *oleifera*)	Increased plant tolerance to Cr, Mn, Co, Cu and Zn<br>Plant growth promotion	Organic degradation and hyphae-accumulation of heavy metals<br>IAA production<br>ACC desaminase activity<br>P solubilization	Roots / no mechanism identified	Zahoor et al. (2017)
Neosartorya sp.	Pakchoi or Chinese cabbage (*B. campestris* var. *chinensis/pekinensis*; *B. rapa* var. *chinensis/pekinensis*)	Plant growth promotion	GAs production	Roots / no mechanism identified	Hamayun et al. (2011)
Nigrospora sp.	Cauliflower (*B. oleracea* var. *botrytis*)	Increased resistance to *S. litura* damage	Systemic defenses activation	Roots / no mechanism identified	Thakur et al. (2013)
Paraphoma radicina	Horseradish (*A. rusticana*)	-	-	Roots /GSLs degradation by fungus	Szűcs et al. (2018)
Penicillium sp.	Rapeseed (*B. napus*)	Increased phytoextraction and plant-tolerance to Cd and Pb<br>Plant growth promotion	Siderophores production<br>IAA production<br>P solubilization	Roots / no mechanism identified	Shi et al. (2017)
Phialocephala fortinii	Pakchoi or Chinese cabbage (*B. campestris* var. *chinensis/pekinensis*; *B. rapa* var. *chinensis/pekinensis*)	Increased disease resistance against protista *P. brassicae*	Unidentified	Roots / no mechanism identified	Narisawa et al. (2004)
Phialocephala sp.	Kale (*B. oleracea* var. *acephala*)	Plant tolerance under cold stress<br>Increased disease resistance against bacteria *X. campestris* pv. *campestris*	Systemic defenses activation	Roots / no mechanism identified	Poveda et al. (2020)
Pseudogymnoascus pannorum	Rapeseed (*B. napus*)<br>Cabbage (*B. oleracea* var. *capitata*)<br>Turnip (*B. rapa*)	Reduced disease caused by fungus *Leptosphaeria maculans*	Unidentified	Roots / no mechanism identified	Roodi et al. (2021)
Pseudosigmoidea ibarakiensis	Pakchoi or Chinese cabbage (*B. campestris* var. *chinensis/pekinensis*; *B. rapa* var. *chinensis/pekinensis*)	Increased phytoextraction and plant-tolerance to Cs	Unidentified	Roots / no mechanism identified	Diene et al. (2014)
Pyrenophora gallaeciana	Kale (*B. oleracea* var. *acephala*)	Plant growth promotion<br>Plant tolerance under cold stress	Unidentifed	Roots / no mechanism identified	Poveda et al. (2020)
Ramichloridium cerophilum	Pakchoi or Chinese cabbage (*B. campestris* var. *chinensis/pekinensis*; *B. rapa* var. *chinensis/pekinensis*)	Plant growth promotion	Unidentified	Roots / no mechanism identified	Ling et al. (2016)
Sclerotinia sclerotiorum	Rapeseed (*B. napus*)	Plant growth promotion<br>Increased disease resistance against fungus *S. sclerotiorum*	Induction of genes related to plant growth and defenses	Endophytic roots colonization by strain infected by a mycovirus	Zhang et al. (2020)
Sebacina vermifera	Cabbage (*B. oleracea* var. *capitata*)<br>Rapeseed (*B. napus*)	Plant growth promotion	Unidentified	Roots / no mechanism identified	Dolatabadi and Mohammadi Goltapeh (2013)

ENDOPHYTIC FUNGUS	BRASSICACEAE CROP	EFFECT	MECHANISM OF ACTION	TISSUE COLONIZATION/ MODE	REFERENCES
	Black mustard (*B. nigra*)<br>Cress (*L. sativum*)<br>Hoary stock (*M. incana*)<br>Flixweed (*D. sophia*)				
Serendipita indica (=*Piriformospora indica*)	Pakchoi or Chinese cabbage (*B. campestris* var. *chinensis/pekinensis*; *B. rapa* var. *chinensis/pekinensis*)	Plant tolerance under drought stress	Increased plant antioxidant activity<br>Induction of genes related to tolerance to drought<br>Increased CAS protein in leaves	Roots / no mechanism identified	Sun et al. (2010)
	Pakchoi or Chinese cabbage (*B. campestris* var. *chinensis/pekinensis*; *B. rapa* var. *chinensis/pekinensis*)	Plant growth promotion	Auxins root synthesis of induction	Roots / no mechanism identified	Lee et al. (2011)
	Cabbage (*B. oleracea* var. *capitata*)<br>Rapeseed (*B. napus*)<br>Black mustard (*Brassica nigra*)<br>Cress (*Lepidium sativum*)<br>Hoary stock (*Matthiola incana*)<br>Flixweed (*Descurainia sophia*)	Plant growth promotion	Unidentified	Roots / no mechanism identified	Dolatabadi and Goltapeh (2013)
	Pakchoi or Chinese cabbage (*B. campestris* var. *chinensis/pekinensis*; *B. rapa* var. *chinensis/pekinensis*)	Improved leaves-nutritional quality	Induction of leaves accumulation of nutraceutical metabolites	Roots / no mechanism identified	Khalid et al. (2017)
	Broccoli (*B. oleracea* var. *italica*)	Plant growth promotion	Unidentified	Roots / no mechanism identified	Singhal et al. (2017)
	Rapeseed (*B. napus*)	Promoted plant growth, seed yield and quality	Increased nutrients supply<br>Repression of erucic acid biosynthesis genes	Roots / no mechanism identified	Su et al. (2017)
	Pakchoi or Chinese cabbage (*B. campestris* var. *chinensis/pekinensis*; *B. rapa* var. *chinensis/pekinensis*)	Plant tolerance under salinity stress	Increased plant antioxidant activity<br>Induction of genes and hormonal pathways related to tolerance to salinity	Roots / no mechanism identified	Khalid et al. (2018)
	Rapeseed (*B. napus*)	Plant growth promotion<br>Increased nutrients content in plant-tissues	Induced phosphatase activity in roots	Roots / no mechanism identified	Wu et al. (2018)
	Pakchoi or Chinese cabbage (*B. campestris* var. *chinensis/pekinensis*; *B. rapa* var. *chinensis/pekinensis*)	Increased disease resistance against protista *P. brassicae*	Local defenses activation	Roots / no mechanism identified	Khalid et al. (2020)

ENDOPHYTIC FUNGUS	BRASSICACEAE CROP	EFFECT	MECHANISM OF ACTION	TISSUE COLONIZATION/ MODE	REFERENCES
Setophoma terrestris	Horseradish (*A. rusticana*)	-	-	Roots/ GSLs degradation by fungus	Szűcs et al. (2018)
Setophoma terrestris *Simplicillium lamellicola*	Kale (*B. oleracea* var. *acephala*)	Increased disease resistance against bacteria *X. campestris* pv. *campestris* Increased resistance to *M. brassicae* damage	Systemic defenses activation	Roots / no mechanism identified	Poveda et al. (2020)
	Rapeseed (*B. napus*)	Antagonism against fungus *S. sclerotiorum*	Mycoparasitism or competition for space and nutrients	Roots / no mechanism identified	Zhang et al. (2014)
Trichoderma atroviride	Mustard (*Brassica juncea* var. *foliosa*)	Increased phytoextraction and plant-tolerance to Cd and Ni	Organic degradation of heavy metals	Roots / no mechanism identified	Cao et al. (2008)
Trichoderma atroviride *T. citrinoviride*	Pakchoi or Chinese cabbage (*B. campestris* var. *chinensis/pekinensis*; *B. rapa* var. *chinensis/pekinensis*)	Plant growth promotion	IAA production Siderophores production K solubilization	Roots / no mechanism identified	Chen et al. (2021)
	Pakchoi or Chinese cabbage (*B. campestris* var. *chinensis/pekinensis*; *B. rapa* var. *chinensis/pekinensis*)	Plant growth promotion	IAA production Siderophores production K solubilization	Roots / no mechanism identified	Chen et al. (2021)
T. hamatum	Kale (*B. oleracea* var. *acephala*) Cabbage (*B. oleracea* var. *capitata*) Leaf rape (*B. napus* var. *napus*) Turnip tops and greens (*B. rapa* var. *rapa*)	Plant growth promotion Improved leaves-nutritional quality	Induction of leaves accumulation of nutraceutical metabolites	Roots / no mechanism identified	Velasco et al. (2021)
T. harzianum	Turnip (*B. rapa*)	Increased resistance to disease against nematode *Meloidogyne incognita*	Unidentified	Unidentified	Ibrahim et al. (2012)
T. harzianum *T. koningiopsis*	Indian mustard (*B. juncea*)	Plant tolerance under salinity stress	Induced plant-antioxidant activity	Roots / no mechanism identified	Ahmad et al. (2015)
	Cauliflower (*B. oleracea* var. *botrytis*)	Increased plant-tolerance to Pb	Increased plant antioxidant activity	Roots / no mechanism identified	Afshari et al. (2018)
	Rapeseed (*B. napus*)	Increased yield	Unidentified	Roots/ Plant myrosinase activity inhibition by fungal myrosinase-binding protein THKEL1, reducing the formation of toxic compounds from GSLs hydrolisis	Poveda et al. (2019a)
	Rapeseed (*B. napus*)	Increased yield	Unidentified	Roots/ The reduction of SA-related local defenses is necessary for fungal root colonization	Poveda et al. (2019b)
	Rapeseed (*B. napus*)	Plant tolerance under salinity and drought stresses	Unidentified	Roots / no mechanism identified	Poveda (2020)

ENDOPHYTIC FUNGUS	BRASSICACEAE CROP	EFFECT	MECHANISM OF ACTION	TISSUE COLONIZATION/ MODE	REFERENCES
	Cauliflower (*B. oleracea* var. *botrytis*)	*Delia radicum* larvae mortality	Parasitism	Roots, stems and leaves/ no mechanism identified	Razinger et al. (2018)
T. parareesei	Rapeseed (*B. napus*)	Plant tolerance under salinity and drought stresses	Induction of genes and hormonal pathways related to tolerance to salinity and drought	Roots / no mechanism identified	Poveda (2020)
Trichoderma sp.	Indian mustard (*B. juncea*)	Increased phytoextraction and plant-tolerance to Cd and Ni	Unidentified	Roots / no mechanism identified	Jiang et al. (2008)
Trichoderma sp.<br>*Veronaeopsis simplex*	Kale (*B. oleracea* var. *acephala*)	Increased disease resistance against bacteria *X. campestris* pv. *campestris*	Systemic defenses activation	Roots / no mechanism identified	Poveda et al. (2020)
	Pakchoi or Chinese cabbage (*B. campestris* var. *chinensis/pekinensis*; *B. rapa* var. *chinensis/pekinensis*)	Reduced disease caused by fungus *F. oxysporum*	Competition for space<br>Siderophores production<br>Local defenses activation<br>Modification of diversity and abundance of rhizosphere fungi	Roots / no mechanism identified	Khastini et al. (2012)
Veronaeopsis simplex<br>*Verticillium* sp.	Pakchoi or Chinese cabbage (*B. campestris* var. *chinensis/pekinensis*; *B. rapa* var. *chinensis/pekinensis*)	Increased phytoextraction and plant-tolerance to Cs	Unidentified	Roots / no mechanism identified	Diene et al. (2014)
	Cauliflower (*B. oleracea* var. *botrytis*)	Increased disease resistance against fungus *Verticillium longisporum*	Competition for space<br>Local defenses activation	Roots / no mechanism identified	Tyvaert et al. (2014)
Unidentified	Field mustard (*B. campestris*; *B. rapa* var. *oleifera*)	Plant growth promotion	N and P supply	Roots / no mechanism identified	Maulana et al. (2021)

References

Afshari, H., Esmaeeli, V., and Khazaee, M. (2018). Effects of various concentrations of *Trichoderma harzianum* fungus on the phytochemical and antioxidative properties of cauliflower (*Brassica oleracea*. convar. *botrytis* L.) in the soils contaminated with lead. *J. Nutr. Fasting Heal.* 6, 35–44. doi:10.22038/jnfh.2018.33579.1123.

Ahmad, P., Hashem, A., Abd-Allah, E. F., Alqarawi, A. A., John, R., Egamberdieva, D., et al. (2015). Role of *Trichoderma harzianum* in mitigating NaCl stress in Indian mustard (*Brassica juncea* L) through antioxidative defense system. *Front. Plant Sci.* 6, 868. doi:10.3389/FPLS.2015.00868/BIBTEX.

Auer, S., and Ludwig-Müller, J. (2014). Effects of the endophyte *Acremonium alternatum* on oilseed rape (*Brassica napus*) development and clubroot progression. *Albanian J. Agric. Sci*. Available at: https://www.researchgate.net/publication/263214054 [Accessed March 30, 2022].

Batta, Y. A. (2013). Efficacy of endophytic and applied *Metarhizium anisopliae* (Metch.) Sorokin (Ascomycota: Hypocreales) against larvae of *Plutella xylostella* L. (Yponomeutidae: Lepidoptera) infesting *Brassica napus* plants. *Crop Prot.* 44, 128–134. doi:10.1016/J.CROPRO.2012.11.001.

Cao, L., Jiang, M., Zeng, Z., Du, A., Tan, H., and Liu, Y. (2008). *Trichoderma atroviride* F6 improves phytoextraction efficiency of mustard (*Brassica juncea* (L.) Coss. var. *foliosa* Bailey) in Cd, Ni contaminated soils. *Chemosphere* 71, 1769–1773. doi:10.1016/J.CHEMOSPHERE.2008.01.066.

Chen, D., Hou, Q., Jia, L., and Sun, K. (2021). Combined use of two *Trichoderma* strains to promote growth of pakchoi (*Brassica chinensis* L.). *Agronomy* 11, 726. doi:10.3390/AGRONOMY11040726.

Deng, Z., Cao, L., Huang, H., Jiang, X., Wang, W., Shi, Y., et al. (2011). Characterization of Cd- and Pb-resistant fungal endophyte *Mucor* sp. CBRF59 isolated from rapes (*Brassica chinensis*) in a metal-contaminated soil. *J. Hazard. Mater.* 185, 717–724. doi:10.1016/J.JHAZMAT.2010.09.078.

Deng, Z., Zhang, R., Shi, Y., Hu, L., Tan, H., and Cao, L. (2013). Enhancement of phytoremediation of Cd- and Pb-contaminated soils by self-fusion of protoplasts from endophytic fungus *Mucor* sp. CBRF59. *Chemosphere* 91, 41–47. doi:10.1016/J.CHEMOSPHERE.2012.11.065.

Diene, O., Sakagami, N., and Narisawa, K. (2014). The role of dark septate endophytic fungal isolates in the accumulation of cesium by chinese cabbage and tomato plants under contaminated environments. *PLoS One* 9, e109233. doi:10.1371/JOURNAL.PONE.0109233.

Dolatabadi, H. K., and Mohammadi Goltapeh, E. (2013). Effect of inoculation *with Piriformospora indica* and *Sebacina vermifera* on growth of selected Brassicaceae plants under greenhouse conditions. *J. Hortic. Res.* 21, 115–124. doi:10.2478/johr-2013-0030.

Dugassa-Gobena, D., Raps, A., and Vidal, S. (1998). Influence of fungal endophytes on allelochemicals of their host plants and the behaviour of insects. *Meded. Landbouwkd. en Toegepaste Biol. Wet. Univ. Gent* 63, 333–337.

Hamayun, M., Khan, S. A., Khan, A. L., Ahmad, N., Nawaz, Y., Sher, H., et al. (2011). Gibberellin producing *Neosartorya* sp. CC8 reprograms Chinese cabbage to higher growth. *Sci. Hortic. (Amsterdam).* 129, 347–352. doi:10.1016/J.SCIENTA.2011.03.046.

Ibrahim, A., Shahid, A. A., Shafiq, M., and Haider, M. S. (2012). Management of root knot nematodes on the turnip plant (*Brassica rapa*) by using fungus (*Trichoderma harzianum*) and neem (*Azadirachta indica*) and effect on the growth rate. *J. Phytopathol* 24, 101–105.

Jiang, M., Cao, L., and Zhang, R. (2008). Effects of Acacia (*Acacia auriculaeformis* A. Cunn)-associated fungi on mustard (*Brassica juncea* (L.) Coss. var. foliosa Bailey) growth in Cd- and Ni-contaminated soils. *Lett. Appl. Microbiol.* 47, 561–565. doi:10.1111/J.1472-765X.2008.02454.X.

Khalid, M., Hassani, D., Bilal, M., Liao, J., and Huang, D. (2017). Elevation of secondary metabolites synthesis in *Brassica campestris* ssp. *chinensis* L. via exogenous inoculation of Piriformospora indica with appropriate fertilizer. *PLoS One* 12, e0177185. doi:10.1371/JOURNAL.PONE.0177185.

Khalid, M., Hassani, D., Liao, J., Xiong, X., Bilal, M., and Huang, D. (2018). An endosymbiont Piriformospora indica reduces adverse effects of salinity by regulating cation transporter genes, phytohormones, and antioxidants in *Brassica campestris* ssp. *chinensis*. *Environ. Exp. Bot.* 153, 89–99. doi:10.1016/J.ENVEXPBOT.2018.05.007.

Khalid, M., Hui, N., Rahman, S.-, Hayat, K., and Huang, D. (2020). Suppression of clubroot (*Plasmodiophora brassicae*) development in *Brassica campestris* sp. *chinensis* L. via exogenous inoculation of *Piriformospora indica*. *J. Radiat. Res. Appl. Sci.* 13, 180–190. doi:10.1080/16878507.2020.1719337.

Khastini, R. O., and Jannah, R. (2021). Potential contribution of dark-septate endophytic fungus isolated from Pulau Dua Nature Reserve, banten on growth promotion of Chinese cabbage. *Jt. Proc. 2nd 3rd International Conf. Food Secur. Innov. (ICFSI 2018-2019)* 9, 83–89. doi:10.2991/absr.k.210304.015.

Khastini, R. O., Ohta, H., and Narisawa, K. (2012). The role of a dark septate endophytic fungus, *Veronaeopsis simplex* Y34, in Fusarium disease suppression in Chinese cabbage. *J. Microbiol. 2012 504* 50, 618–624. doi:10.1007/S12275-012-2105-6.

Lee, Y.-C., Johnson, J. M., Chien, C.-T., Sun, C., Cai, D., Lou, B., et al. (2011). Growth promotion of Chinese cabbage and Arabidopsis by *Piriformospora indica* is not stimulated by mycelium-synthesized auxin. *Mol. Plant-Microbe Interact.* 24, 421–431. doi:10.1094/MPMI-05-10-0110.

Ling, X., Erika, U., and Kazuhiko, N. (2016). A endophytic fungus, *Ramichloridium cerophilum*, promotes growth of a non-mycorrhizal plant, Chinese cabbage. *African J. Biotechnol.* 15, 1299–1305. doi:10.5897/AJB2016.15398.

Maulana, A. F., Turjaman, M., Hashimoto, Y., Cheng, W., and Tawaraya, K. (2021). Nitrogen and phosphorus concentrations in growth media affect the relationship between root endophytic fungi and host plant. *Arch. Microbiol.* 203, 2411–2418. doi:10.1007/S00203-021-02238-1/FIGURES/3.

Moisan, K., Dicke, M., Raaijmakers, J. M., Rachmawati, E., and Cordovez, V. (2021). Volatiles from the fungus *Fusarium oxysporum* affect interactions of *Brassica rapa* plants with root herbivores. *Ecol. Entomol.* 46, 240–248. doi:10.1111/EEN.12956.

Morita, S., Azuma, M., Aoba, T., Satou, H., Narisawa, K., and Hashiba, T. (2003). Induced systemic resistance of Chinese cabbage to bacterial leaf spot and Alternaria leaf spot by the root endophytic fungus, *Heteroconium chaetospira*. *J. Gen. Plant Pathol. 2003 691* 69, 71–75. doi:10.1007/S10327-002-0005-Z.

Narisawa, K., Ohki, K. T., and Hashiba, T. (2000). Suppression of clubroot and Verticillium yellows in Chinese cabbage in the field by the root endophytic fungus, *Heteroconium chaetospira*. *Plant Pathol.* 49, 141–146. doi:10.1046/j.1365-3059.2000.00425.x.

Narisawa, K., Shimura, M., Usuki, F., Fukuhara, S., and Hashiba, T. (2005). Effects of pathogen density, soil moisture, and soil ph on biological control of clubroot in Chinese cabbage by *Heteroconium chaetospira*. *Plant Dis.* 89, 285–290. doi:10.1094/PD-89-0285.

Narisawa, K., Tokumasu, S., and Hashiba, T. (1998). Suppression of clubroot formation in Chinese cabbage by the root endophytic fungus, *Heteroconium chaetospira*. *Plant Pathol.* 47, 206–210. doi:10.1046/J.1365-3059.1998.00225.X.

Narisawa, K., Usuki, F., and Hashiba, T. (2004). Control of Verticillium yellows in Chinese cabbage by the dark septate endophytic fungus LtVB3. *Phytopathology*® 94, 412–418. doi:10.1094/PHYTO.2004.94.5.412.

Poveda, J. (2020). *Trichoderma parareesei* favors the tolerance of rapeseed (*Brassica napus* L.) to salinity and drought due to a chorismate mutase. *Agronomy* 10, 118. doi:10.3390/agronomy10010118.

Poveda, J., Hermosa, R., Monte, E., and Nicolás, C. (2019a). The *Trichoderma harzianum* Kelch protein ThKEL1 plays a key role in root colonization and the induction of systemic defense in Brassicaceae plants. *Front. Plant Sci.* 10, 1478. doi:10.3389/FPLS.2019.01478/BIBTEX.

Poveda, J., Hermosa, R., Monte, E., and Nicolás, C. (2019b). *Trichoderma harzianum* favours the access of arbuscular mycorrhizal fungi to non-host Brassicaceae roots and increases plant productivity. *Sci. Reports 2019 91* 9, 1–11. doi:10.1038/s41598-019-48269-z.

Poveda, J., Zabalgogeazcoa, I., Soengas, P., Rodríguez, V. M., Cartea, M. E., Abilleira, R., et al. (2020). *Brassica oleracea* var. *acephala* (kale) improvement by biological activity of root endophytic fungi. *Sci. Reports 2020 101* 10, 1–12. doi:10.1038/s41598-020-77215-7.

Qin, J., Lyu, A., Zhang, Q. hua, Yang, L., Zhang, J., Wu, M. de, et al. (2019). Strain identification and metabolites isolation of *Aspergillus capensis* CanS-34A from *Brassica napus*. *Mol. Biol. Rep.* 46, 3451–3460. doi:10.1007/S11033-019-04808-5/TABLES/3.

Raps, A., and Vidal, S. (1998). Indirect effects of an unspecialized endophytic fungus on specialized plant – herbivorous insect interactions. *Oecologia 1998 1144* 114, 541–547. doi:10.1007/S004420050478.

Razinger, J., Lutz, M., Grunder, J., and Urek, G. (2018). Laboratory investigation of cauliflower–fungus–insect interactions for cabbage maggot control. *J. Econ. Entomol.* 111, 2578–2584. doi:10.1093/JEE/TOY228.

Roodi, D., Millner, J. P., McGill, C. R., Johnson, R. D., Hea, S. Y., Brookes, J. J., et al. (2021). Development of Plant–Fungal endophyte associations to suppress Phoma stem canker in *Brassica*. *Microorg. 2021, Vol. 9, Page 2387* 9, 2387. doi:10.3390/MICROORGANISMS9112387.

Shi, Y., Xie, H., Cao, L., Zhang, R., Xu, Z., Wang, Z., et al. (2017). Effects of Cd- and Pb-resistant endophytic fungi on growth and phytoextraction of *Brassica napus* in metal-contaminated soils. *Environ. Sci. Pollut. Res.* 24, 417–426. doi:10.1007/S11356-016-7693-Y/TABLES/5.

Singhal, U., Khanuja, M., Prasad, R., and Varma, A. (2017). Impact of synergistic association of ZnO-Nanorods and symbiotic fungus *Piriformospora indica* DSM 11827 on *Brassica oleracea* var. *botrytis* (Broccoli). *Front. Microbiol.* 8, 1909. doi:10.3389/FMICB.2017.01909/BIBTEX.

Su, Z., Wang, T., Shrivastava, N., Chen, Y., Liu, X., Sun, C., et al. (2017). *Piriformospora indica* promotes growth, seed yield and quality of Brassica napus L. *Microbiol. Res.* 199, 29–39. doi:10.1016/j.micres.2017.02.006.

Sun, C., Johnson, J. M., Cai, D., Sherameti, I., Oelmüller, R., and Lou, B. (2010). *Piriformospora indica* confers drought tolerance in Chinese cabbage leaves by stimulating antioxidant enzymes, the expression of drought-related genes and the plastid-localized CAS protein. *J. Plant Physiol.* 167, 1009–1017. doi:10.1016/j.jplph.2010.02.013.

Szűcs, Z., Plaszkó, T., Cziáky, Z., Kiss-Szikszai, A., Emri, T., Bertóti, R., et al. (2018). Endophytic fungi from the roots of horseradish (*Armoracia rusticana*) and their interactions with the defensive metabolites of the glucosinolate - myrosinase - isothiocyanate system. *BMC Plant Biol.* 18, 1–15. doi:10.1186/S12870-018-1295-4/FIGURES/6.

Thakur, A., Kaur, S., Kaur, A., and Singh, V. (2013). Enhanced Resistance to Spodoptera litura in Endophyte Infected Cauliflower Plants. *Environ. Entomol.* 42, 240–246. doi:10.1603/EN12001.

Tyvaert, L., França, S. C., Debode, J., and Höfte, M. (2014). The endophyte *Verticillium* Vt305 protects cauliflower against Verticillium wilt. *J. Appl. Microbiol.* 116, 1563–1571. doi:10.1111/JAM.12481.

Usuki, F., and Narisawa, K. (2017). A mutualistic symbiosis between a dark septate endophytic fungus, *Heteroconium chaetospira*, and a nonmycorrhizal plant, Chinese cabbage. *http://dx.doi.org/10.1080/15572536.2007.11832577* 99, 175–184. doi:10.1080/15572536.2007.11832577.

Velasco, P., Rodríguez, V. M., Soengas, P., and Poveda, J. (2021). *Trichoderma hamatum* increases productivity, glucosinolate content and antioxidant potential of different leafy *Brassica*

vegetables. *Plants 2021, Vol. 10, Page 2449* 10, 2449. doi:10.3390/PLANTS10112449.

Worapong, J., and Strobel, G. A. (2009). Biocontrol of a root rot of kale by Muscodor albus strain MFC2. *BioControl* 54, 301–306. doi:10.1007/S10526-008-9175-8/TABLES/3.

Wu, M., Wei, Q., Xu, L., Li, H., Oelmüller, R., and Zhang, W. (2018). *Piriformospora indica* enhances phosphorus absorption by stimulating acid phosphatase activities and organic acid accumulation in *Brassica napus*. *Plant Soil* 432, 333–344. doi:10.1007/S11104-018-3795-2/FIGURES/5.

Zahoor, M., Irshad, M., Rahman, H., Qasim, M., Afridi, S. G., Qadir, M., et al. (2017). Alleviation of heavy metal toxicity and phytostimulation of Brassica campestris L. by endophytic *Mucor* sp. MHR-7. *Ecotoxicol. Environ. Saf.* 142, 139–149. doi:10.1016/J.ECOENV.2017.04.005.

Zhang, H., Xie, J., Fu, Y., Cheng, J., Qu, Z., Zhao, Z., et al. (2020). A 2-kb mycovirus converts a pathogenic fungus into a beneficial endophyte for *Brassica* protection and yield enhancement. *Mol. Plant* 13, 1420–1433. doi:10.1016/J.MOLP.2020.08.016.
